# Mechanistic Identification of Oxygen Species in the Degradation of CsPbBr_3_ Quantum Dot Films Through Real-Time In Situ Monitoring

**DOI:** 10.3390/ma18235467

**Published:** 2025-12-04

**Authors:** Zewen Lin, Jie Song, Haixia Wu, Hongliang Li, Rui Huang

**Affiliations:** School of Physics and Electronic Engineering, Hanshan Normal University, Chaozhou 521041, China; zewenlin@126.com (Z.L.); songjie@hstc.edu.cn (J.S.); 20220063@hstc.edu.cn (H.W.);

**Keywords:** oxygen species, CsPbBr_3_ QD films, photoluminescence, degradation

## Abstract

**Highlights:**

**What are the main findings?**

- Neutral O_2_ does not alter the emission or structure of CsPbBr_3_ QD films even under UV illumination.
- Reactive oxygen species (ROS) cause rapid PL quenching and lifetime shortening.
- ROS create Br vacancies and Pb–O bonds, generating deep nonradiative traps.

**What are the implications of the main findings?**

- Oxygen-induced degradation originates from activated oxygen, not molecular O_2_.
- Plasma processing conditions must be carefully controlled to avoid ROS damage.
- Strategies such as passivation and encapsulation can preserve perovskite stability.

**Abstract:**

The chemical identity of oxygen species plays a decisive role in determining the optical stability of halide perovskite QD films. Here, real-time in situ spectroscopic monitoring, together with steady-state and time-resolved photoluminescence measurements, is utilized to differentiate the effects of molecular oxygen and plasma-activated oxygen species on CsPbBr_3_ QD films. The films maintain nearly unchanged emission intensity, spectral profile, and carrier lifetimes when stored in vacuum or exposed to molecular O_2_ even under UV illumination, demonstrating that neutral O_2_ exhibits minimal reactivity toward the [PbBr_6_]^4−^ framework. In contrast, oxygen plasma generates highly reactive atomic and ionic oxygen species that induce rapid and spatially heterogeneous photoluminescence quenching. This degradation is attributed to Br^−^ extraction, Br-vacancy formation, and subsequent Pb–O bond generation, which collectively introduce deep trap states and enhance nonradiative recombination. These findings clearly indicate that reactive oxygen species rather than molecular O_2_ are the dominant driver of oxygen-induced luminescence degradation, providing mechanistic insight and offering processing guidelines for the reliable integration of perovskite nanomaterials in optoelectronic devices.

## 1. Introduction

Lead halide perovskites have emerged as a new generation of optoelectronic semiconductors due to their exceptional carrier transport properties, high absorption coefficients, and defect-tolerant electronic structure, enabling rapid progress in light-emitting diodes, solar cells, and photodetectors over the past decade [1,2,3,4,5,6]. However, their practical application remains hindered by intrinsic instability that arises from their soft, ionic lattices. In hybrid perovskites such as MAPbI_3_, the volatility of organic cations (MA^+^ and FA^+^) under light, heat, moisture, and oxygen accelerates irreversible phase decomposition [7]. Compared with hybrid systems, all-inorganic cesium lead halide perovskites (CsPbX_3_, X = Cl, Br, I) show significantly improved thermal and structural stability due to the absence of volatile organic species. Nevertheless, their optical properties still deteriorate under UV illumination, heating, or polar environments [8,9,10]. Considerable efforts have therefore been devoted to improving the stability of CsPbX_3_ through interface engineering and encapsulation strategies, and encouraging progress has been achieved [11,12,13,14,15,16]. Despite these advances, the mechanistic role of oxygen in perovskite degradation remains a topic of debate.

Early studies suggested that molecular O_2_ alone exerts only a minimal effect on the stability of MAPbI_3_, with moisture identified as the dominant factor responsible for ionic loss and phase transformation [7]. In contrast, Bryant et al. observed rapid performance decay of CH_3_NH_3_PbI_3_ devices in dry oxygen under illumination [17]. Subsequent work revealed that oxygen can rapidly diffuse along grain boundaries and halide vacancy sites, where reactive oxygen species form preferentially and induce structural corrosion [18]. More recently, Hidalgo et al. demonstrated a cooperative degradation pathway in which H_2_O dissolves surface organic cations and I^−^ while O_2_ oxidizes the exposed Pb–I framework into iodate species, further weakening the perovskite lattice [19]. In parallel, seemingly contradictory observations have been reported for CsPbBr_3_, where moderate oxygen exposure can temporarily enhance photoluminescence quantum yield (PLQY) [20]. This “photobrightening” has been attributed to weak molecular O_2_ adsorption that passivates deep traps at surface Pb-rich or Br-vacancy sites, reducing nonradiative recombination [21]. However, prolonged illumination drives electron transfer to adsorbed oxygen, generating reactive oxygen radicals that erode the Pb–Br framework, extract halides, and ultimately form deep trap states that accelerate optical degradation [21,22].

In this work, we resolve these conflicting interpretations by systematically examining the role of oxygen species in the luminescence stability of CsPbBr_3_ quantum dot (QD) films through real-time in situ spectroscopic monitoring, complemented by steady-state PL and time-resolved photoluminescence (TRPL) analyses. We find that neutral molecular O_2_ exhibits negligible reactivity toward the CsPbBr_3_ lattice even under UV illumination, while plasma activation generates reactive oxygen species that induce rapid PL quenching, carrier lifetime shortening, and lattice disruption. These results provide direct mechanistic evidence that chemically active oxygen species rather than molecular O_2_ are responsible for oxygen-induced degradation, offering clear guidance for maintaining optical integrity during materials processing and device fabrication.

## 2. Materials and Methods

### 2.1. Preparation of CsPbBr_3_ QD Films and Oxygen Treatments

A colloidal dispersion of CsPbBr_3_ QDs (10 mg mL^−1^ in hexane) was purchased from Nanjing MKNANO Tech. Co., Ltd., Nanjing, China. The QDs were synthesized following the hot-injection protocol developed by Protesescu et al. [1]. The QDs, partially capped with oleic acid and oleylamine ligands, exhibit a PLQY of ~75% and a particle size distribution of 9–11 nm. For film fabrication, the QD solution was spin-coated onto pre-cleaned quartz substrates at 4000 rpm for 30 s, yielding uniform emissive layers.

The as-prepared films were subsequently placed under different oxygen environments, including high vacuum, molecular O_2_, and oxygen plasma. The plasma treatment was conducted in a very-high-frequency plasma-enhanced chemical vapor deposition (VHF-PECVD) system. During plasma exposure, chamber pressure, flow rate and substrate temperature were controlled at ~20 Pa, 20 SCCM and ~30 °C, respectively.

### 2.2. Real-Time In Situ Spectroscopic Monitoring

**Figure 1:**
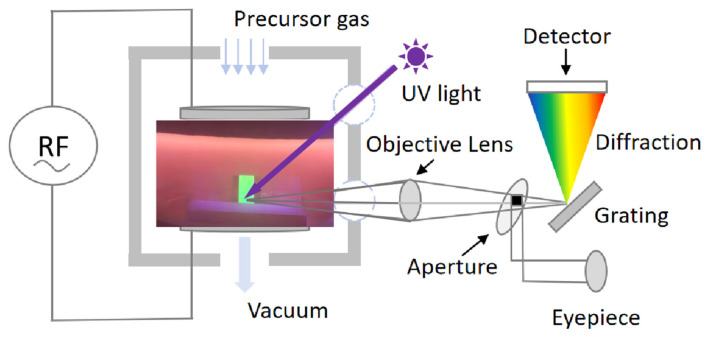
Schematic illustration of the real-time and in situ spectroscopic monitoring setup used to record the optical emission of CsPbBr_3_ QD films in different plasma atmospheres.

In situ luminescence evolution was recorded using a custom diagnostic configuration (schematic shown in Figure 1) in which the VHF-PECVD reactor (self-designed) was directly coupled to a Photo Research PR-655 SpectraScan spectroradiometer (Photo Research Inc., North Syracuse, New York, NY, USA). A 365 nm UV excitation lamp (8 W) was employed to excite the CsPbBr_3_ QD films during the monitoring process, and the optical emission was continuously collected at room temperature. This setup enabled real-time tracking of emission intensity and spectral changes throughout oxygen exposure.

### 2.3. Optical Characterization

Absorption spectra of the CsPbBr_3_ QD films were measured in transmission mode using a Shimadzu UV-3600 spectrophotometer (Shimadzu Corporation, Kyoto, Japan). Steady-state PL and TRPL measurements were performed on an Edinburgh Instruments FLS1000 spectrometer (Edinburgh Instruments Ltd., Livingston, West Lothian, Scotland, UK) at 300 K. A pulsed 375 nm diode laser (pulse width ~70 ps) served as the excitation source. The emitted photons were detected using a photomultiplier tube (PMT) coupled to a monochromator and analyzed through a time-correlated single-photon counting (TCSPC) module, providing a temporal resolution of approximately 50 ± 4 ps. The steady-state PL excitation density (~0.1 mW cm^−2^) and TRPL pulse fluence (~0.05 μJ cm^−2^) were sufficiently low to avoid photobleaching or carrier-induced modification of surface chemistry.

## 3. Results and Discussion

**Figure 2:**
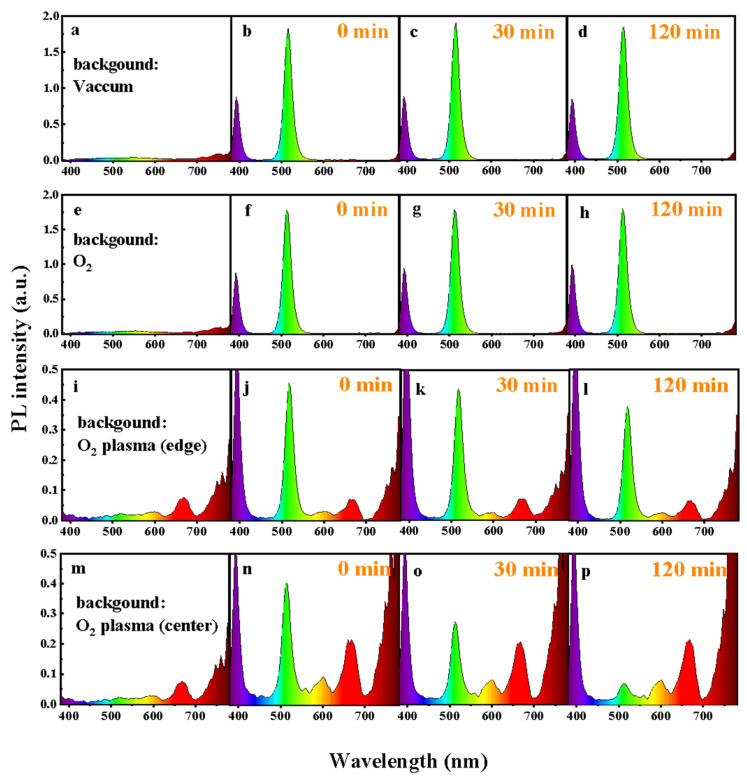
Optical emission spectra for different gas atmosphere and CsPbBr_3_ QD films modified in: (**a**–**d**) vacuum, (**e**–**h**) O_2_, (**i**–**l**) the edge of O_2_ plasma, and (**m**–**p**) the center of O_2_ plasma. During plasma exposure, the RF power, chamber pressure, flow rate and substrate temperature were controlled at 30 W, ~20 Pa, 20 SCCM and ~30 °C, respectively.

**Figure 3:**
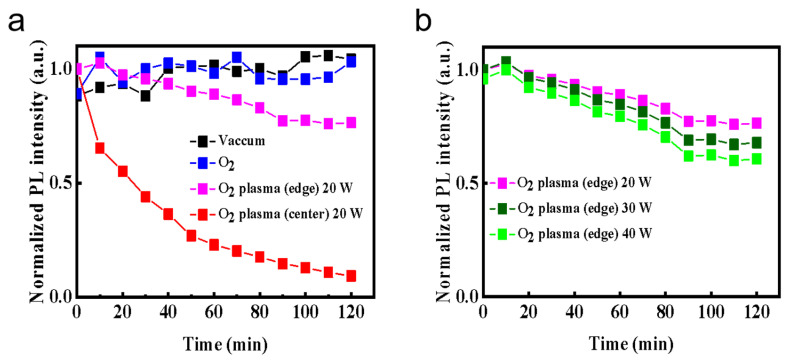
Time-dependent evolution of the normalized PL intensity of CsPbBr_3_ QD films under different oxygen environments (**a**) and O_2_ plasma with different RF powers (**b**).

Figure 1 shows the real-time in situ spectroscopic monitoring system equipped with an optical emission detector. Using this setup, we continuously recorded the PL evolution of CsPbBr_3_ QD films under 365 nm UV excitation, as displayed in Figure 2. The films were measured either in vacuum or in controlled oxygen environments, including molecular O_2_ and O_2_ plasma. For the plasma condition, two regions within the discharge zone were selected: the plasma edge and the plasma center, referred to as “O_2_ plasma (edge)” and “O_2_ plasma (center),” respectively. In addition to in situ PL tracking, optical emission spectra of the oxygen plasma were recorded under the same VHF conditions. As shown in Figure 2i,m, the discharge exhibits clear emission at ~526 nm (O_2_^+^ ionic emission), ~679 nm (excited O*), and ~777 nm (atomic oxygen O, O I transition) [23], confirming that both molecular ionic and atomic oxygen species are present in the plasma at the plasma edge and plasma center. Moreover, under VHF-PECVD conditions, the spatial distribution of reactive oxygen species is expected to vary significantly across the plasma region. At the plasma center, the locally enhanced electron density and strong sheath electric field facilitate electron-impact dissociation of O_2_, leading to elevated generation of O, O*, and O_2_^+^ species. In contrast, at the plasma edge, the sheath potential and ion bombardment are substantially reduced, while neutral ROS efficiently diffuse outward from the plasma bulk, resulting in a lower but still chemically active concentration of reactive oxygen species [24]. As shown in Figure 2, all films exhibit a characteristic CsPbBr_3_ emission peak at ~515 nm. However, the PL stability varies markedly depending on the chemical state of oxygen. Under vacuum, the PL spectra remain nearly unchanged for over 120 min (Figure 2d), confirming the excellent intrinsic stability of the QDs in the absence of reactive species. Similarly, exposure to molecular O_2_ results in negligible changes in PL intensity or spectral shape even under UV illumination (Figure 2f–h), indicating that neutral O_2_ molecules interact weakly with the film surface and do not induce observable degradation. This behavior arises from the absence of moisture, which suppresses the formation of O_2_^−^ species [22]. These results contrast with previous reports showing transient PL enhancement followed by slow oxidative degradation under prolonged illumination, where photoexcited electrons activate adsorbed O_2_ into reactive O_2_^−^ species that attack the Pb–Br framework and create deep trap states [19,20,21,22]. In sharp contrast, exposure to O_2_ plasma causes conspicuous and rapid PL degradation. When the film is positioned at the plasma center (Figure 2n–p), the PL intensity drops by more than 50% within 30 min (Figure 3a). This severe quenching arises from the combined action of chemically active oxygen radicals and physical ion bombardment [22,25,26,27,28], which together induce surface etching, and lattice distortion. These processes create deep nonradiative trap states, leading to significant emission loss. To decouple the chemical oxidation effects from the physical ion-induced damage, the films were also examined at the plasma edge, where ion energy is greatly reduced while reactive oxygen radicals are still present. In this region, the PL decay proceeds much more gradually (Figure 2j–l). As shown in Figure 3a, the PL decreases by ~10% after 30 min and by ~25% after 120 min, confirming that reactive oxygen species alone can drive degradation, though at a slower rate. Moreover, increasing the plasma power at the plasma edge accelerates PL quenching (Figure 3b), further demonstrating that the concentration of reactive oxygen species rather than ion bombardment is the dominant factor governing luminescence degradation. As shown in Figure 2i,m, the oxygen plasma generates species such as O* and O via electron-impact dissociation and excitation. These highly reactive species can extract Br^−^ and oxidize the Pb–Br framework, leading to the formation of Pb–O bonds and deep-level defects that promote nonradiative recombination and accelerate emission decay [8,19]. Collectively, these results clarify that CsPbBr_3_ QD films exhibit long-term luminescence stability in vacuum and molecular O_2_ even under UV illumination, whereas reactive oxygen species are the primary drivers of degradation, with the extent of damage directly correlated to their concentration and activity.

**Figure 4:**
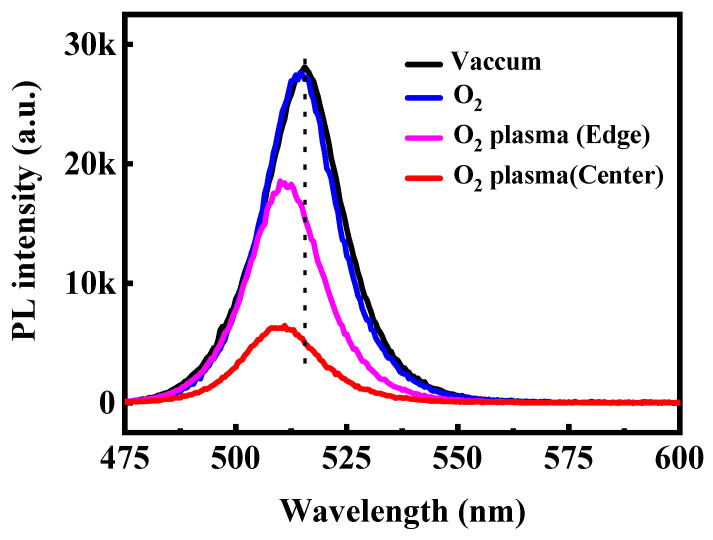
PL spectra of the CsPbBr_3_ QDs treated in the different conditions excited by the 365 nm line from Xe lamp.

**Figure 5:**
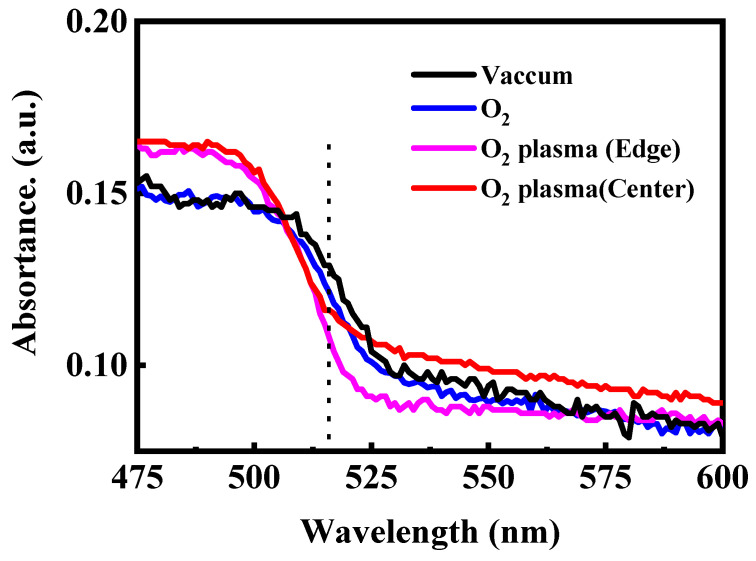
UV–vis absorption spectra of the CsPbBr_3_ QDs treated in the different conditions.

To further verify the impact of oxygen species on the optical stability of CsPbBr_3_ QD films, steady-state PL and UV–vis absorption spectra were recorded after 120 min of exposure to different oxygen atmospheres. As shown in Figure 4, when maintained in vacuum or placed in molecular O_2_, the films retain nearly identical PL intensity and spectral shape, with the emission peak remaining at ~515 nm. The absence of peak shift or intensity loss confirms that neutral O_2_ molecules exert negligible influence on the emissive behavior of CsPbBr_3_ QDs in the absence of activation pathways such as light-driven charge transfer or humidity-assisted reactions. In contrast, O_2_ plasma leads to pronounced PL attenuation. The film located at the plasma edge exhibits a moderate reduction in emission intensity, while the film placed at the plasma center shows severe quenching, with more than two-thirds of the PL intensity lost after 120 min. This spatially dependent degradation reflects the varying densities of reactive oxygen species and energetic ions across the plasma region. We note that the PL quenching induced by O_2_ plasma is irreversible, indicating that the CsPbBr_3_ QD degradation arises from permanent structural and chemical modifications. A noticeable blue shift in the PL peak emerges upon plasma exposure, likely arising from plasma-induced surface etching, which reduces the average particle size and thereby enhances quantum confinement. The UV–vis absorption spectra shown in Figure 5 exhibit a consistent trend. Films preserved in vacuum or exposed to molecular O_2_ show nearly unchanged absorption edges, indicating stable crystal and electronic structures. Conversely, films treated with O_2_ plasma show a shift of the absorption edge toward shorter wavelengths accompanied by a decrease in absorbance intensity. This effect is most prominent at the plasma center, where the flux of reactive oxygen and ions is highest. The coincident blue shift in both PL emission and absorption confirms that reactive oxygen species produced by O_2_ plasma not only induces chemical oxidation but also physically erodes the QD surface, narrowing the effective particle size and widening the optical bandgap.

**Figure 6:**
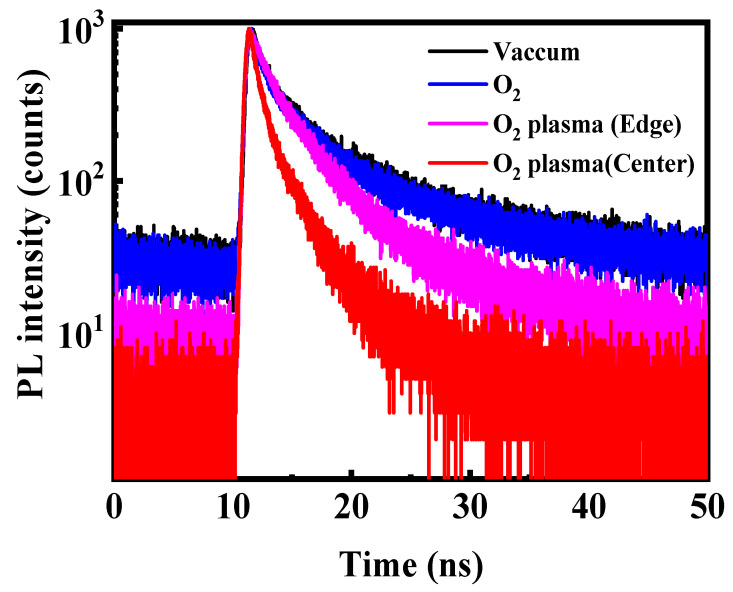
Time-resolved PL decay curves of the CsPbBr_3_ QD films treated in the different conditions excited by the 372 nm pulse laser.

To further resolve the carrier recombination pathways associated with oxygen-induced degradation, TRPL measurements were conducted on CsPbBr_3_ QD films after exposure to different oxygen environments (Figure 6). All decay curves were well described by a bi-exponential function [29]:

R(t) = K_1_exp(−t∕τ_1_) + K_2_exp(−t∕τ_2_) (1)

where K_1_ and K_2_ represent the fractional contributions of fast and slow decay components, and τ_1_ and τ_2_ correspond to their respective lifetimes. The fast component (τ_1_) reflects trap-assisted nonradiative recombination, while the slow component (τ_2_) corresponds to intrinsic radiative recombination within well-passivated domains [30]. The fitted parameters are summarized in Table 1, and the average PL lifetime (τ_ave_) was calculated according to the method described in Ref. [29]. For films stored in vacuum or exposed to molecular O_2_, the decay dynamics remain dominated by the slow radiative component (K_2_ ≈ 86%), with τ_2_ ≈ 7.5–7.6 ns and an average lifetime of ~6.7 ns. The nearly unchanged τ_1_, τ_2_, and K_1_/K_2_ ratios indicate that neutral O_2_ molecules do not introduce additional trap states nor disrupt the lattice, consistent with the steady-state PL and absorption results. This stability originates from the chemical inertness of ground-state O_2_, whose strong O=O bond prevents spontaneous halide abstraction or Pb^2+^ oxidation in the absence of activation energy. In contrast, O_2_ plasma treatment leads to a pronounced acceleration of carrier decay. The film located at the plasma center exhibits the shortest τ_1_ (0.8 ns) and the highest contribution of the fast component (K_1_ = 49.0%), signifying the emergence of abundant nonradiative recombination sites. Such behavior results from the combined chemical and physical impact of reactive oxygen radicals and energetic ions, which disrupt surface passivation, remove ligands, and distort the Pb–Br framework. At the plasma edge, where ion bombardment is significantly mitigated but reactive oxygen radicals remain present, τ_1_ increases to 1.3 ns and K_1_ decreases to 23.5%. This intermediate behavior confirms that reactive oxygen species alone are sufficient to form trap states, albeit more slowly.

**Figure 7:**
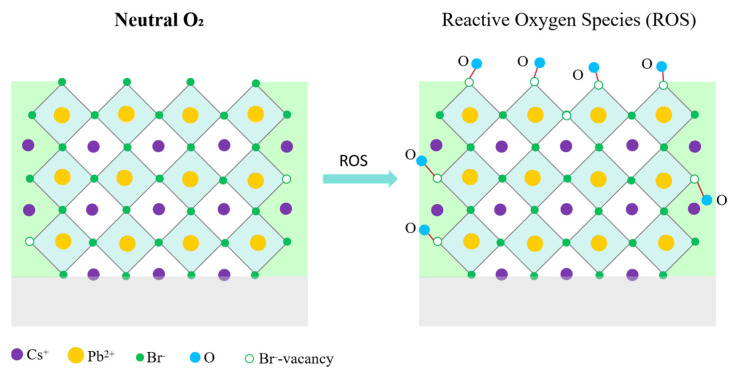
Schematic mechanism distinguishing the inert effect of molecular O_2_ and the degradative role of reactive oxygen species in CsPbBr_3_ QD films.

Thus, the collective TRPL, steady-state PL, and absorption results establish a clear mechanistic distinction between neutral O_2_ molecules and activated oxygen species in determining the optical stability of CsPbBr_3_ QDs, as is shown in Figure 7. Molecular O_2_, possessing a strong O=O bond and limited surface reactivity, does not disrupt the [PbBr_6_]^4−^ coordination environment even under photoactivated conditions and therefore preserves long-lived radiative recombination. In contrast, oxygen plasma produces highly reactive species (O, O*, and O_2_^+^) capable of abstracting Br^−^ and creating Br vacancies (V_Br_), which in turn facilitate the formation of Pb–O bonds and lattice distortion [8,19]. These structural perturbations introduce deep trap states and significantly increase nonradiative recombination pathways, consistent with the accelerated τ_1_ and increased K_1_ components observed in TRPL analysis. Thus, the luminescence degradation of CsPbBr_3_ QDs originates from chemically active oxygen species rather than neutral molecular oxygen even under photoactivated conditions.

## 4. Conclusions

In summary, we have elucidated the distinct roles of neutral molecular oxygen and plasma-activated reactive oxygen species in governing the optical stability of CsPbBr_3_ QD films through real-time in situ monitoring combined with steady-state and time-resolved spectroscopies. Under illumination, neutral O_2_ shows negligible interaction with the [PbBr_6_]^4−^ lattice, and both photoluminescence intensity and carrier lifetime remain stable. In contrast, oxygen plasma generates reactive oxygen species (O, O*, and O_2_^+^) that extract Br^−^, create halide vacancies, and induce Pb–O bond formation, leading to an increase in deep trap states and enhanced nonradiative recombination. These processes result in rapid luminescence degradation. This work identifies activated oxygen species rather than molecular O_2_ as the true origin of oxygen-induced instability. We note that the current experimental setup does not permit quantitative determination of ROS concentration or flux at different plasma positions. Developing calibrated ROS profiling to distinguish chemical oxidation from ion-induced effects, and extending these insights to device-relevant environments and other perovskite compositions, will be important directions for future work. Overall, the mechanistic insights gained here offer practical implications for plasma processing, surface passivation, and encapsulation strategies to enhance the reliability of halide perovskite-based optoelectronic devices.

## Figures and Tables

**Table 1:** Summary of the fitting parameters for the PL decay traces of the CsPbBr_3_ QD films treated in the different conditions.

Sample	τ_1_/ns	K_1_	τ_2_/ns	K_2_	Τ_ave._/ns
O_2_	1.5 ± 0.2	13.9 ± 2.0%	7.5 ± 1.0	86.1 ± 2.0%	6.7
Vacuum	1.5 ± 0.3	14.1 ± 2.5%	7.6 ± 1.5	85.9 ± 2.5%	6.7
O_2_ plasma (Edge)	1.3 ± 0.2	23.5 ± 3.0%	6.1 ± 1.2	76.5 ± 3.0%	5.0
O_2_ plasma (Center)	0.8 ± 0.2	49.0 ± 3.0%	5.0 ± 1.0	51.0 ± 3.0%	2.9

## Data Availability

The raw data supporting the conclusions of this article will be made available by the authors on request.
